# Study on the Curing Behaviors of Benzoxazine Nitrile-Based Resin Featuring Fluorene Structures and the Excellent Properties of Their Glass Fiber-Reinforced Laminates

**DOI:** 10.3390/ma17246167

**Published:** 2024-12-17

**Authors:** Mingzhen Xu, Lunshuai He, Jiaqu Zhang, Zexu Fan, Bo Li

**Affiliations:** School of Materials and Energy, University of Electronic Science and Technology of China, Chengdu 610054, China; helunshuai123@163.com (L.H.); 19908094739@163.com (J.Z.); fanzexu150@163.com (Z.F.); uestc_lb87@163.com (B.L.)

**Keywords:** benzoxazine, fluorene, fiber-reinforced laminates, mechanical properties, thermal stability

## Abstract

Benzoxazine and o-phthalonitrile resin are two of the most eminent polymer matrices within high-performance fiber-reinforced resin-based composite materials. Studying the influence modalities of their structures and forming processes on performance can furnish a theoretical basis for the design and manufacturing of superior performance composite materials. In this study, we initially incorporated a fluorene structure into the molecular main chain through molecular design to prepare a fluorene-containing benzoxazine nitrile-based resin. The polymerization reaction behavior and process of this resin were monitored meticulously using differential scanning calorimetry and infrared spectroscopy. Meanwhile, by manipulating the pre-polymerization reaction conditions, the impact of the pre-polymerization reaction on the polymerization behavior of the resin monomer was investigated, respectively. Subsequently, diverse glass fiber-reinforced resin-based composite materials were fabricated via hot-pressing in combination with a programmed temperature rise process. Through the characterization of structural strength and thermomechanical properties, it was found that the composite laminates all manifested outstanding bending strength (~600 MPa) and modulus (>30 GPa). Nevertheless, with the elevation of the post-curing temperature, the structural strength and modulus of the composite materials displayed distinct variation laws. This study also discussed the variation laws of the thermal properties of the composite materials by analyzing the glass transition temperature and crosslinking density. Additionally, the interface bonding effect between the glass fiber and the resin matrix was deliberated through the analysis of the cross-sectional morphology of the composite laminates. The results demonstrated that this work proposes an improved matrix resin system with outstanding thermal stability and mechanical properties that broadens the foundation and ideas for subsequent research.

## 1. Introduction

Thermosetting resins are a class of polymers that undergo a chemical change when cured, resulting in a compact spatial crosslinked network structure. This unique structural configuration imparts several advantageous properties to thermosetting resins, including high dimensional stability, outstanding thermal resistance, and significant chemical resistance [1,2,3]. These characteristics make them suitable for various applications across multiple industries such as automotive, aerospace, electronics, and construction. Phthalonitrile-based resin containing benzoxazine is a highly significant type of high-performance thermosetting resin [4,5,6,7]. It showcases an array of remarkable characteristics, including outstanding high-temperature resistance, an extremely elevated glass transition temperature, exceptional thermal stability and mechanical properties, an extremely low water absorption rate, superior flame retardancy, and almost negligible shrinkage, among other notable attributes. Simultaneously, it holds several advantageous attributes such as autocatalytic polymerization, convenient formation processes, and the feasibility of structural designability [7]. As a result, it has found extensive application in the manufacturing of resin-based composite materials [8,9,10]. The monomer of phthalonitrile-based resin containing benzoxazine encompasses active functional groups, namely amino and phenolic groups, and follows a distinct two-stage polymerization mechanism [6]. Specifically, under heating conditions, the oxazine ring undergoes ring-opening polymerization to generate the active phenolic Mannich bridge structure. Subsequently, the crosslinking polymerization of nitrile groups is catalyzed, thereby achieving the effect of self-catalytic polymerization [7].

Additionally, benzoxazine resin stands out among thermosetting resins due to its remarkable molecular design flexibility compared to other polymer types. This flexibility allows for the creation of diverse molecular structures tailored to specific performance requirements [11]. The ability to modify the molecular structure is crucial because it directly influences the physical and mechanical properties of benzoxazine resin. For instance, variations in molecular architecture can lead to changes in curing behavior, thermal stability, and overall durability.

Understanding how alterations in molecular structure affect the performance of benzoxazine resin is essential for expanding its applicability into broader fields. Researchers have been actively investigating different modifications that can enhance these materials’ properties. For example, Chen et al. [12] introduced quinoxaline structures into benzoxazine resin formulations; this modification resulted in a notable reduction of 45 °C in the initial curing temperature. Such reductions not only facilitate processing but also improve energy efficiency during manufacturing. In another study by Zhu et al. [13], eugenol was incorporated into benzoxazine resin systems, which significantly improved the thermal stability of the resultant material. Eugenol’s natural phenolic structure contributes additional crosslinking sites within the polymer matrix while enhancing heat resistance, which is an important factor for applications requiring prolonged exposure to elevated temperatures. Furthermore, fluorene and its derivatives represent another category of compounds with rigid planar biphenyl structures that have shown promise in modifying resins effectively. Tan et al. [14] explored this avenue by introducing fluorene groups during polyimide polymer synthesis processes; their findings indicated that synthesized polyimide resins exhibited excellent thermal stability alongside impressive dimensional integrity characterized by a glass transition temperature (*T_g_*) reaching up to 370 °C and an exceptionally low coefficient of thermal expansion (CTE) measured at 5.72 ppm/°C.

In previous studies, the benzoxazine nitrile-based resin monomer containing a fluorene structure was successfully synthesized through a series of well-defined chemical reactions [15]. The variations in activation energy of the resin during the curing process were meticulously investigated both before and after introducing the fluorene structure. Furthermore, a self-catalytic model of the reaction was developed alongside an analysis of alterations in kinetic parameters such as rate constants and reaction order. These models are essential for predicting how modifications at a molecular level can influence macroscopic properties like viscosity and gelation time during processing. Through comprehensive calculations of activation energy derived from experimental data, it was discovered that incorporating the fluorene structure significantly increased steric hindrance within the resin monomer framework [16]. This increase in steric bulk not only affected individual molecular interactions but also led to substantial changes in crosslinked network structures formed during resin curing processes. Such structural modifications are crucial because they directly impact the mechanical strength, thermal stability, and overall performance characteristics of cured resins.

With the advancement of science and technology, particularly in material sciences, there is an escalating demand for diversification in the materials field to meet specific application requirements across different industries [17,18,19]. Single-component thermosetting resins have become inadequate to satisfy these increasingly intricate application demands due to limitations such as thermal stability, mechanical strength, and resistance to environmental factors. The modification of resin matrices through fiber reinforcement represents an effective approach to enhance both performance characteristics and broaden application scopes significantly. Glass fiber (GF) stands out among various reinforcing agents due to its exceptional properties that make it suitable for demanding applications [19]. As a high-performance inorganic fiber with a working temperature exceeding 300 °C coupled with favorable mechanical performance—evidenced by tensile modulus values surpassing 52 GPa—it also exhibits remarkable corrosion resistance against harsh environments. Consequently, glass fiber has been extensively employed in research focused on enhancing both structural integrity and functional capabilities within high-performance resin matrices.

For instance, Xu et al. [20] fabricated innovative high-performance resin-based composite materials that demonstrated impressive bending strengths exceeding 550 MPa alongside glass transition temperatures above 450 °C by incorporating glass fibers into phthalonitrile resins. Similarly, Ratan et al. [21] utilized glass fibers effectively for reinforcing epoxy resins; their efforts resulted in composite materials characterized by outstanding mechanical properties—including bending moduli and strengths greater than 450 MPa—and impact strengths reaching beyond 1350 J/m. These composites are essential for fulfilling stringent requirements found in high-end application domains like aerospace engineering, automotive manufacturing, marine construction (shipbuilding), electronics packaging, and other sectors where durability and reliability are paramount.

According to the literature [15], FAEN-Bz resin was subjected to heat treatment under various temperature conditions, namely 220 °C, 240 °C, 260 °C, and 280 °C, for 2 h at each temperature to investigate the effect of the curing degree on the final properties. Firstly, the relationship between the curing temperature and the phase morphology was studied. SEM images of poly(FAEN-Bz) treated at 260 °C and 280 °C demonstrated that the phase morphology evolved with the increase in the curing temperature. After the treatment at 260 °C, the ring-opening polymerization of benzoxazine rings resulted in a relatively homogeneous phase state. After the treatment at 280 °C, the ring-opening polymerization of benzoxazine rings was nearly complete, and the images showed a homogeneous phase state consisting mainly of Mannich bridge and aromatic ester nitrile segments arranged orderly. Then, the relationship between the curing temperature and the crosslinking degree was discussed. The crosslinking degree of poly(FAEN-Bz) cured at 260 °C and 280 °C was 85.0% and 96.3%, respectively, indicating that the resin cured at 280 °C has a relatively high crosslinking degree. In our recent work [22], benzoxazine containing nitrile-resin with a fluorene structure (WZ-cn) was heat-treated at various temperatures (200 °C, 220 °C, 240 °C) to obtain high-performance polymers. The results revealed that the phase morphology and the crosslinking degree of the resin changed significantly with the rise in the curing temperature. Particularly under higher temperatures, the ring-opening polymerization of benzoxazine rings was almost complete, resulting in a highly homogeneous phase state and a high degree of crosslinking (96.3%). These findings are crucial for understanding the effect of the curing temperature on the properties of benzoxazine-based resin composites.

Building upon this prior research, a kind of benzoxazine nitrile-based resin featuring fluorene structures (WZ-cn) was designed and prepared. Then, solution impregnation combined with hot-pressing techniques was designed for fabricating the glass fiber-reinforced WZ-cn composite laminates (WZ-cn/GF). In this study, we deliberated on the effect of the curing temperature on the properties of benzoxazine-based resin. The curing reaction process of WZ-CN was investigated through the transformation of functional groups subsequent to curing at various temperatures. By comparing the thermal stability and mechanical properties of the WZ-cn/GF composite laminates at different curing temperatures (200 °C, 240 °C, and 280 °C), we discovered that the thermal stability of the composites was affected significantly by the curing temperatures. Additionally, we also observed that the tensile and bending strength of the wound composite improved under a higher curing regime. These results emphasize the significance of suitable curing temperatures for optimizing the properties of composites and enhancing the overall performance. Moreover, this work delves into exploring variation laws concerning property changes within these composite materials following different post-curing temperature treatments, which play a crucial role in determining final material characteristics such as hardness or flexibility based on intended use cases or operational environments encountered during service life cycles. Subsequently, a systematic assessment was undertaken focusing on critical aspects including interfacial interaction dynamics—which influence load transfer efficiency—thermomechanical behavior under varying thermal conditions (thermal expansion coefficients), comprehensive mechanical property evaluations (tensile strength tests), along with assessments related to thermal stability attributes necessary for ensuring longevity.

## 2. Materials and Methods

### 2.1. Raw Materials

The phthalonitrile-based resin containing benzoxazine based on bisphenol fluorene (WZ-cn), formaldehyde, and 3-aminophenoxyl-o-phthalonitrile was synthesized with 1, 4-dioxide, and methylbenzene as the solvent. Bisphenol fluorene and formaldehyde were obtained from Chengdu Kelong chemicals Co. Ltd., Chengdu, China. 1, 4-dioxane and methylbenzene were purchased from Shanghai Bodi chemical Co. Ltd., Shanghai, China. Aminophenoxy phthalonitrile (APN, *T_m_* = 174 °C) was prepared and purified in our lab [23]. All reagents were of analytical grade and used without further purification.

### 2.2. Preparation of WZ-cn Monomers

**Figure 1:**
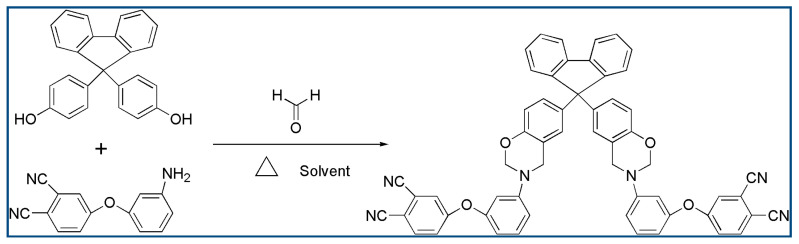
The synthesis process of the WZ-cn monomer.

The phthalonitrile-based resin containing benzoxazine based on bisphenol fluorene (WZ-cn) was prepared by using bisphenol fluorene, formaldehyde, and 3-aminophenoxyl-o-phthalonitrile as raw materials, and 1, 4-dioxane and methylbenzene as the solvent (volume ratio 10:1). In a three-necked flask, firstly, bisphenol fluorene, formaldehyde, and 3-aminophenoxyl-o-phthalonitrile with the molar ratio of 1:4:2 were dissolved in 1, 4-dioxane solvent to obtain a brown–yellow suspension, and then toluene was added into the mixed solution. The mixed solution was heated slowly to 85~100 °C for 6 h under the condition of mechanical stirring and reflux condensation. The water and toluene were removed by a water separator. Then, the obtained products were poured into cold deionized water to precipitate. Finally, the obtained products were washed with hot water and filtered several times, and then dried in an oven at 80 °C. A purified purplish brown–yellow solid powder was obtained (yield: 95%). The synthetic route is shown in Figure 1 and its structure was characterized by ^1^H-NMR and FTIR spectrum.

^1^H-NMR (the proton nuclear magnetic resonance spectrum, 400 MHz, deuterated DMSO) δ(ppm): 4.48 ppm (N-CH_2_-Ar), 5.29 ppm (N-CH_2_-O), 6.62~6.74 (N-Ar-H), 6.88~6.91, and 7.57~7.61(H-Ar-CN).

### 2.3. Preparation of WZ-cn Pre-Polymers

The pre-polymers of WZ-cn were fabricated via the melt approach. The pre-polymerization temperature was fixed at 160 °C and diverse pre-polymerization durations (30 min, 60 min, and 90 min) were selected as variables. The WZ-cn samples with distinct pre-polymerization times were denoted as WZ-cn-30, WZ-cn-60, and WZ-cn-90, respectively. The detailed preparation protocol is exemplified by the manufacture of WZ-cn-60. WZ-cn powder was placed in a clean tin foil box and inserted into a forced-air oven. The temperature was set at 160 °C. Once the oven temperature attained the set value, the timing initiated and it was sustained for 60 min. After removal, the sample was ground into powder for testing and subsequent experiments.

### 2.4. Preparation of Fiber-Reinforced WZ-cn Composite (WZ-cn/GF) Laminates

WZ-cn/GF composite laminates were prepared by the hot-pressing lamination method. First, 10 layers of GFs (after heat treatment, GF: 10 cm × 10 cm, 1.9 g/layer) were prepared. The mass ratio of WZ-cn pre-polymers to GFs was designed to be 45 wt% WZ-cn and 55 wt% GFs. Then, WZ-cn pre-polymers were diluted with DMF to a suitable viscosity and evenly impregnated on the GFs. The obtained prepreg was hung in a fume hood for at least 12 h and subsequently placed in a forced-air oven at 80 °C for 30 min. Subsequently, the prepreg was placed in the forced-air oven and underwent a pretreatment program comprising 160 °C for 20 min and 180 °C for 10 min to obtain a semi-cured laminate. The semi-cured laminate was placed in a stainless steel mold in groups of 10 layers and subjected to a stacking hot-pressing process with a constant pressure of 20 MPa. The heat-press program was set as shown in Table 1 to obtain the cured WZ-cn/GF composite laminates. In contrast, the bisphenol A benzoxazine-based resin (BA-ph) was selected and the glass fiber-reinforced composite laminates (BA-ph/GF) were fabricated under the same process conditions. WZ-cn/GF composite laminates were tailored for mechanical properties and DMA tests.

### 2.5. Characterizations

The proton nuclear magnetic resonance spectrum (^1^H-NMR) was obtained by the nuclear magnetic resonance spectrometer Bruker AV400 (NMR, Bruker, Karlsruhe, Germany) with deuterated DMSO as solvent at a proton frequency of 400 MHz. The Fourier Transform Infrared Spectra (FTIR) was recorded on the FTIR 8400S (Shimadzu, Kyoto, Japan) Fourier Transform Infrared Spectrometer in KBr pellets between 4000 and 400 cm^−1^ in air. Differential scanning calorimetric (DSC) analysis was performed by using a modulated DSC-Q100 (TA Instruments, New Castle, DE, USA) under a nitrogen atmosphere, with a heating rate of 10 °C/min and a constant nitrogen flow rate of 50 mL/min. DSC testing of WZ-cn pre-polymers was performed by heating the samples from 50 °C to 350 °C. The gel chromatographic instrument Rid-20A, manufactured by Shimadzu Company in Japan, was utilized for Gel Permeation Chromatography (GPC) analysis. A sample powder weighing 150 mg was taken and tetrahydrofuran (THF) was chosen as the mobile phase. The sample was completely dissolved in the mobile phase prior to testing. Gelation time was tested by using a gel time tester (YJ39-LA38-11BN, Shanghai, China) of Shanhai Yijia Electric Co., Ltd. Thermogravimetric analysis (TGA) was performed by using TGA-Q50 (TA Instruments, New Castle, DE, USA). In the nitrogen atmosphere, the temperature is increased from 50 °C to 600 °C, the heating rate is 20 °C/min, and the purge rate is 40 mL/min. The mechanical properties were investigated by using the SANS series microcomputer-controlled electronic universal testing machine (CMT6104, Shenzhen, China) using the three-point bending test mode. WZ-cn/GF composite laminates need to be tailored into strips with a length and width of about 50 mm × 10 mm, and the support span to thickness ratio is 15:1. In three-point bending mode, a test speed of 5 mm min 1 for crosshead displacement was used. And the results were obtained by averaging three samples (refer to Chinese standard GB/T 2567-2008) [24]. Dynamic mechanical analysis (DMA) was performed by using a DMA-Q800 (TA Instruments, New Castle, DE, USA) dynamic mechanical analyzer. The WZ-cn/GF composite laminate samples (dimensions 40 mm × 10 mm × 1.5 mm) were heated from 50 °C to 350 °C at a heating rate of 5 °C/min and tested in the three-point bending mode. The morphology of the fracture surface of the material was observed with a scanning electron microscope (SEM) phenom pharos G2 (Feiner, Amsterdam, The Netherlands) at a voltage of 20 kV.

## 3. Results and Discussion

### 3.1. Curing Behaviors of Various WZ-cn Pre-Polymers

The performance of thermosetting resins is highly correlated with the curing process. Pre-polymerization is a frequently employed procedure in the application of thermosetting resins, and it is conducted to accommodate specific processing conditions and processing equipment or to attain the desired performance. Changes in the pre-polymerization conditions have an extensive influence on the curing performance, chemical structure, etc., of the synthetic resin [25].

**Figure 2:**
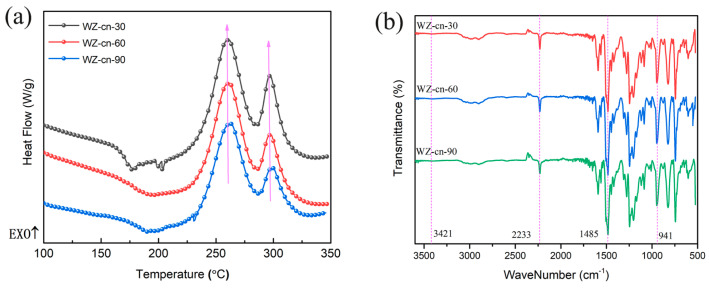
(**a**) DSC curves and (**b**) FTIR spectrum of various WZ-cn pre-polymers.

The curing behaviors of WZ-cn pre-polymers with different pre-polymerization times were investigated by DSC testing. Figure 2a presents the DSC curves of WZ-cn pre-polymers with various pre-polymerization times (30 min, 60 min, and 90 min) at 160 °C. Table 2 lists the corresponding DSC data, respectively: *T_i_* (initial curing temperature), *T_p_*_1_ (peak temperature of the first curing peak), *T_p_*_2_ (peak temperature of the second curing peak), ΔH_1_ (enthalpy of the first curing reaction), and ΔH_2_ (enthalpy of the second curing reaction). As previously mentioned, the first curing reaction is the ring-opening polymerization of the benzoxazine ring, and the second curing reaction is the ring-opening polymerization of the nitrile group [7,26]. According to Table 2, the *T_i_*, *T_p_*_1_, *T_p_*_2_, ΔH_1_, and ΔH_2_ values of the WZ-cn monomer pre-polymer are all lower than those of the pre-polymers. This can be ascribed to the fact that the polymerization reaction of the WZ-cn monomer is primarily initiated by the presence of trace active phenol hydroxyl groups in the system, while the subsequent polymerization process is mainly catalyzed by both phenol hydroxyl groups and imine structures within the Mannich bridge structure formed through oxazine ring-opening [27,28]. The *T_i_* values of the pre-polymers decrease as the pre-polymerization time increases while being higher than that of the WZ-cn monomers. This is because the introduction of the fluorene structure increases the steric hindrance and simultaneously reduces the density of the benzoxazine ring, enhancing the difficulty of the curing reaction. Then, with the increase in pre-polymerization time, the ring-opening reaction of the benzoxazine ring was promoted, and the phenolic hydroxyl groups released from the ring-opening polymerization of the benzoxazine monomer had a catalytic effect on the subsequent polymerization. Meanwhile, due to the reduction in benzoxazine functional groups caused by the prolongation of the pre-polymerization time, the enthalpy of the pre-polymerization reaction also decreases with the extension of the pre-polymerization time. Thus, the ΔH_1_ value of the WZ-cn pre-polymer decreases from 72.90 J/g to 62.41 J/g, and the ΔH_2_ value decreases from 21.32 J/g to 13.35 J/g.

By means of infrared spectroscopy tests, the transformation of functional groups in WZ-cn monomer before and after pre-polymerization treatment can be monitored, as depicted in Figure 2b. It can be discerned from the figure that following the pre-polymerization treatment of WZ-cn, the characteristic absorption peak of the nitrile group at 2230 cm^−1^ does not exhibit significant alterations, signifying that the ring-formation reaction of -CN has not taken place. Previous studies have also verified that the ring-opening polymerization of the nitrile group at 160 °C is scarcely achievable [29]. Meanwhile, the intensity of the characteristic absorption peak of the benzoxazine ring in the vicinity of 951 cm^−1^ diminishes, while the characteristic peak of the Mannich bridge at 1420 cm^−1^ augments, suggesting that the ring-opening reaction of the benzoxazine ring has occurred and, after opening, it mainly forms the Mannich bridge structure in the form of phenolic hydroxyl groups [27,28]. Combined with the DSC test results, it can be expounded that, on the one hand, the ring-opening reaction of the benzoxazine ring in WZ-cn is rather arduous, resulting in a lower content of generated hydroxyl groups. Secondly, due to the considerable steric hindrance of the main chain structure, the phenolic hydroxyl groups formed by the ring-opening of the oxazine ring cannot freely shift and are more involved in the formation of intramolecular or intermolecular hydrogen bonds, making them hard to detect in the infrared spectrum [30,31].

### 3.2. The Pre-Polymerization Degree and the Gelation Time of Various WZ-cn

**Figure 3:**
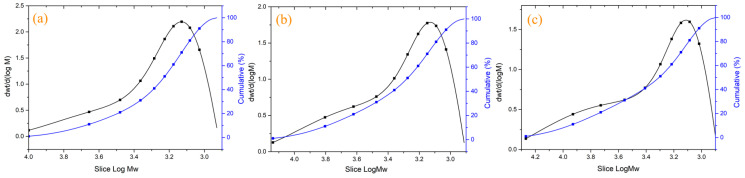
The chromatograms of various WZ-cn pre-polymers: (**a**) WZ-cn-30, (**b**) WZ-cn-60, and (**c**) WZ-cn-90.

To further explore the influence of the pre-polymerization reaction on the polymerization reaction behaviors of WZ-cn, the molecular weights were characterized through GPC tests. By conducting a comparative analysis of the molecular weights and their distribution magnitudes, the facilitating effects of the pre-polymerization conditions and treatment on the ring-opening and ring-forming reactions were clarified. GPC tests were carried out on the WZ-cn pre-polymer, and the obtained results are presented in Figure 3 and Table 3, where *M_z_* denotes the mass-average molecular weight, *M_n_* denotes the number-average molecular weight, *M_w_* represents the weight-average molecular weight, and Dispersity Đ is the dispersity index. With the prolongation of the pre-polymerization treatment time, the *M_z_, M_n_*, and *M_w_* of the WZ-cn pre-polymers rose. According to the variation in *M_n_*, it can be noticed that the WZ-cn monomer formed dimers after the pre-polymerization treatment, which is also in line with the rule of the changes in infrared functional groups.

Meanwhile, as the degree of pre-polymerization escalates, the Dispersity Đ of both pre-polymers also ascends. This is attributed to the fact that the prolonged pre-polymerization treatment gives rise to the formation of polymers with substantial molecular weight disparities and enhances the heterogeneity of the molecular structure within the pre-polymers [30]. Additionally, the degree of Dispersity Đ alteration in the WZ-cn pre-polymer is conspicuous. This could be owing to the introduction of rigid functional groups, which impedes the flexibility of the molecular chain and active functional groups. Consequently, the probability and opportunity of polymerization reactions between active functional groups during the polymerization process decline, causing an uneven internal reaction within the system and manifesting phenomena such as broadened molecular weight distribution and uneven molecular weight distribution.

It is known from the above-mentioned test characterizations that the fluorene-containing benzoxazine cyanate resin treated with different pre-polymerization times have all undergone ring-opening polymerization reactions, but no ring-forming of the cyano groups has occurred. To further explore the polymerization rate of different benzoxazine cyanate resins after pre-polymerization treatment, the gelation test method was employed to test the gelation time of different structural resin systems at 200 °C, and the results are shown in Table 4. It can be observed that the pre-polymerization treatment can shorten the gelation time of the resin system, that is, increase the reaction rate of the resin. The gelation time of WZ-cn pre-polymerized for 90 min is reduced by 154 s compared to that of WZ-cn pre-polymerized for 30 min. This phenomenon is due to the fact that after the pre-polymerization treatment, some oxazine rings undergo ring-opening polymerization, releasing more active phenolic hydroxyl groups. The presence of active groups further promotes the ring-opening of the oxazine rings and even triggers the ring polymerization of the cyano groups, which macroscopically manifests as an acceleration of the polymerization rate of the resin system [7,30,31].

### 3.3. The Time Sweep and Structural Transformation of the WZ-cn Pre-Polymer

**Figure 4:**
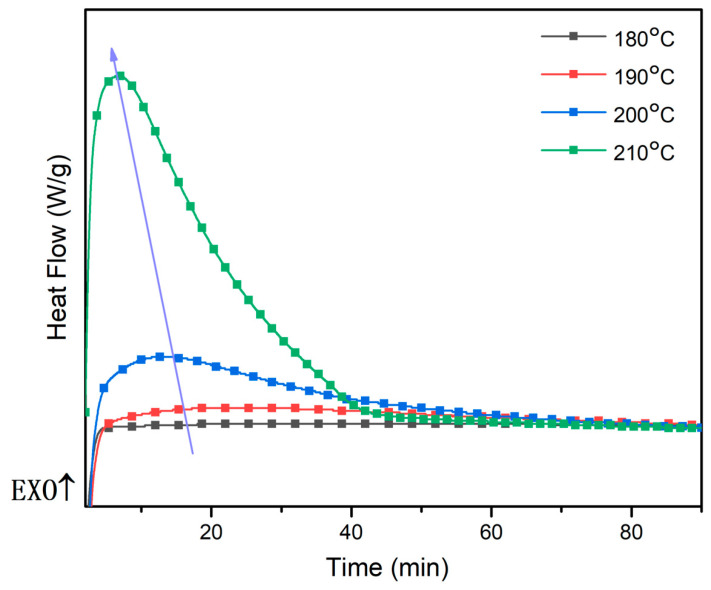
DSC time sweep of the WZ-cn-90 pre-polymer.

When performing the thermal reaction behavior analysis through DSC testing, apart from determining the relationship between heat enthalpy and temperature via temperature scanning, the time sweep test under constant temperature conditions is typically utilized to obtain the variation law of the polymerization degree of the resin over time under different temperature conditions. Herein, the DSC time sweep approach was adopted to monitor the reaction behavior of the WZ-cn-90, and the test results are depicted in Figure 4. Based on the deduced and analyzed outcomes determined by the previous molding process, we initially chose the test temperatures of 180 °C, 190 °C, 200 °C, and 210 °C. It can be noted from Figure 4 that a distinct exothermic peak for WZ-cn did not emerge until 200 °C and the exothermic peak had a large and gentle time span; when the test temperature rose to 210 °C, there remained a single broad and gentle exothermic peak in the test curve, indicating that the WZ-cn-90 demonstrated a relatively mild and continuous polymerization reaction within this test temperature range. Based on the foregoing analysis, there are two reactions in the benzoxazine cyanate resin system: the ring-opening polymerization of the benzoxazine ring and the ring-forming polymerization of the cyano group [6,7,20,26]. Therefore, it can be inferred from the above test results that at higher temperatures, the ring-opening reaction of the benzoxazine ring proceeds intensely and prefers local self-polymerization. As the degree of polymerization increases, the difficulty of self-polymerization between benzoxazine rings gradually rises, and subsequently, the ring-forming polymerization of the cyano group mainly takes place. This staged polymerization reaction also leads to a separation phenomenon in the variation in heat enthalpy with time during the test, manifested as a double exothermic peak in the time sweep curve. When the test temperature is lower than 210 °C, only a single broad and gentle exothermic peak appears in the scan curve, indicating that the ring-opening polymerization of the benzoxazine ring and the ring-forming polymerization of the cyano group can proceed continuously under relatively low-temperature conditions. Meanwhile, it can also be discovered that the higher the heat treatment temperature, the higher and sharper the peak, and the curing reaction at this time is more intense. In contrast, the lower the heat treatment temperature, the lower the peak height and the wider the peak width. A wider and gentler peak indicates that the post-curing process of the pre-polymer at this temperature is more mild and continuous, and the corresponding polymerization reaction will also be more sufficient [7].

**Figure 5:**
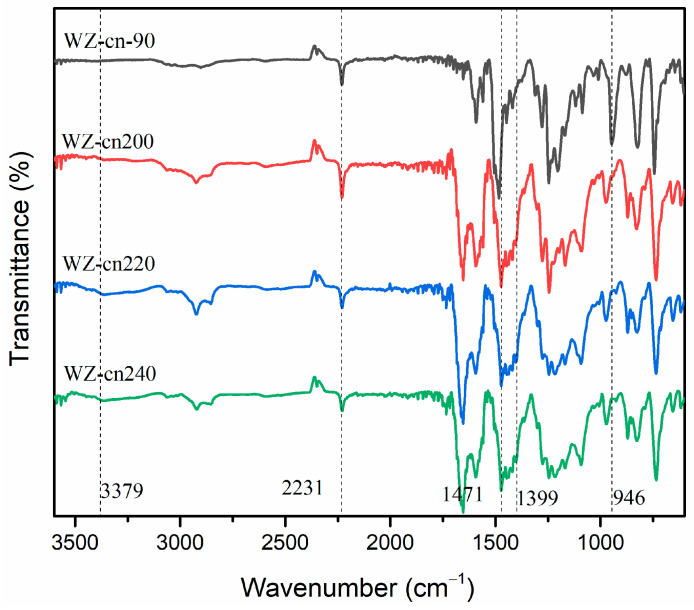
FTIR spectrum of the WZ-cn-90 pre-polymer cured at various temperatures.

The changes in the functional groups of the pre-polymer and various structural benzoxazine nitrile-based resins after undergoing diverse thermal polymerization treatments were monitored and characterized through infrared spectroscopy, and the outcomes are presented in Figure 5. After the post-curing process, the intensity of the characteristic absorption peak of the benzoxazine ring around 953 cm^−1^ decreased, while that of the characteristic peak of the Mannich bridge around 1478 cm^−1^ increased, signifying that the benzoxazine ring opened to form the Mannich bridge structure [30]. The stretching vibration peak of the phenolic hydroxyl group around 3395 cm^−1^ also rose with the formation of the Mannich bridge structure. The absorption peak around 2234 cm^−1^ corresponds to the characteristic absorption peak of -CN, which weakened as the curing degree increased. The characteristic absorption peak around 1395 cm^−1^ is the characteristic absorption peak of the phthalocyanine ring after the curing of the nitrile group, which emerged when the curing began, indicating that WZ-cn underwent ring polymerization of the nitrile group upon curing at the prescribed temperature conditions [26]. It was also discovered that the characteristic absorption peak of the nitrile group still remained in the polymer after the thermal curing treatment at 240 °C, suggesting that the ring-forming polymerization of nitrile groups was incomplete at this temperature. This is mainly because there are a considerable number of rigid aromatic ring structures in the benzoxazine nitrile-based resin system [23,29]. With the growth in the degree of polymerization and crosslinking, the entanglement and steric hindrance among molecular chains increase sharply, making it challenging for the active groups in the main chain and end groups to continue undergoing polymerization reactions, ultimately leaving some active groups. In combination with the literature report [32], it was found that the presence of the nitrile group could still be detected in the phthalocyanine resin polymer after the post-curing treatment at 350 °C, indicating that achieving complete crosslinking of the nitrile group is highly difficult. Under the premise of energy conservation, emission reduction, and green manufacturing, to enhance the universality of the processing technology, we can strive to identify the optimal solution between the polymerization degree and comprehensive performance of the high-performance resin system, allowing for the presence of a small number of nitrile groups in the polymer system.

**Figure 6:**
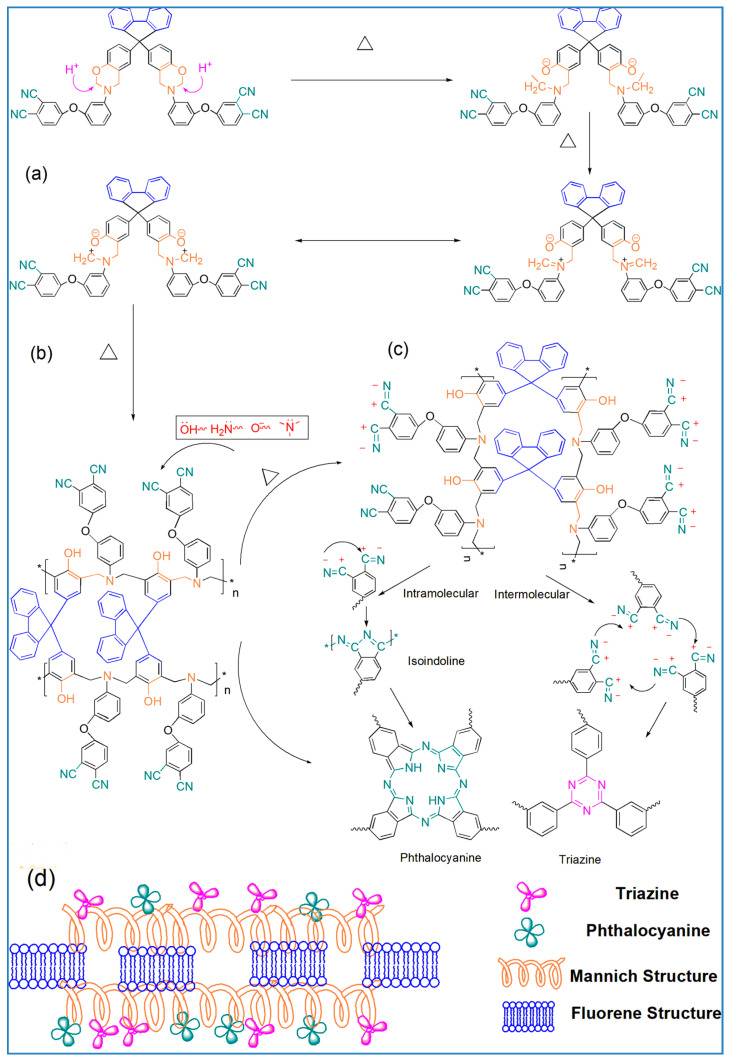
(**a**,**b**) The free hydrogen catalyzes the ring-opening polymerization of the oxazine ring in WZ-cn to form the Mannich bridge structure; (**c**) the active group in the Mannich bridge catalyzes the ring-forming polymerization of the nitrile group in WZ-cn to form a triazine ring and a phthalocyanine ring; and (**d**) possible structure of the WZ-cn polymer.

Consequently, the polymerization reaction process of the WZ-cn might comprise the following stages, addressed in Figure 6. Firstly, a small quantity of free and active hydrogen in the system catalyzed the ring-opening reaction of benzoxazine rings, which occurred at approximately 160 °C (Figure 6a,b) [20,25,26]. The benzoxazine ring mainly forms a Mannich bridge structure with the phenol structure under heat treatment, which is rich in phenolic hydroxyl and imine structures. This structure can further facilitate the ring-opening reaction of oxazine rings and gradually trigger the cyclization polymerization of nitrile groups to form a structure containing aromatic heterocyclic rings such as triazine rings and phthalocyanine rings, mainly taking place at temperatures higher than 220 °C (Figure 6c) [31]. Subsequently, with the increase in temperature and the extension of heat treatment time, the WZ-cn resin can form a polymer with a three-dimensional network structure containing abundant aromatic heterocycles (Figure 6d).

### 3.4. Thermal Properties of WZ-cn/GF Composite Laminates

#### 3.4.1. Thermal Stability of WZ-cn/GF Composite Laminates

**Figure 7:**
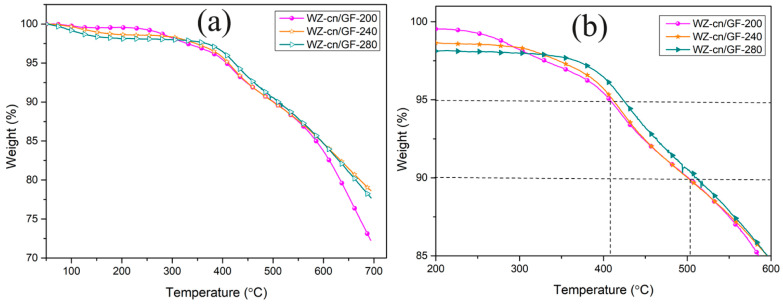
The TGA analysis curves of WZ-cn/GF composite laminates treated at various temperatures: (**a**) TGA; (**b**) zoom into view.

**Figure 8:**
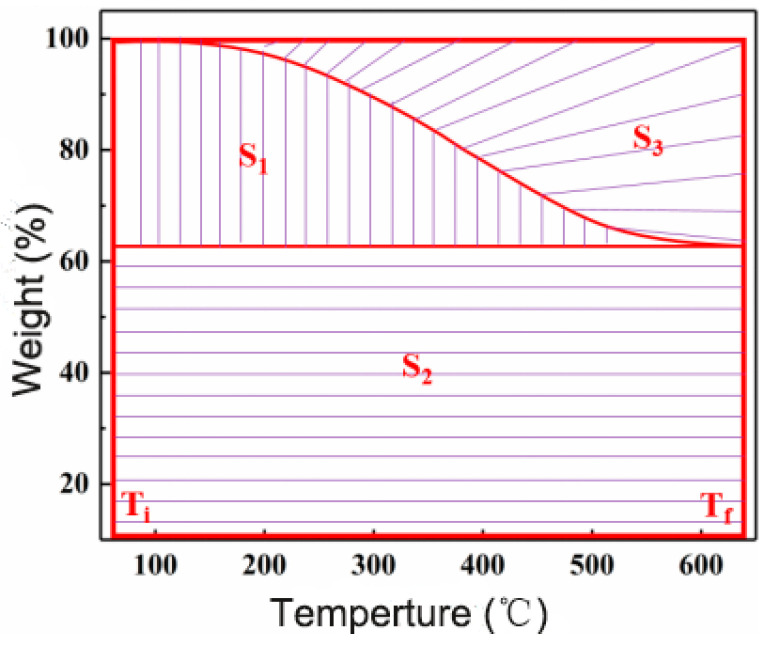
Schematic diagram of S1, S2, and S3 in the formulas A* and K*.

The thermal stability of WZ-cn/GF composites treated at various temperatures was evaluated by the TGA analysis curve. The treatment temperature was 200 °C, 240 °C, and 280 °C, respectively. The TGA curve is shown in Figure 7, and the detailed thermal performance data are shown in Table 5, including the temperature with thermal weight loss of 5% (*T*_5%_) and 10% (*T*_10%_), the residual carbon rate at 600 °C (*Yc*), and the integral program decomposition temperature (IPDT). Among them, IPDT is related to volatile compounds in polymer materials, and its results are not affected by the morphology, particle size, and experimental conditions of the sample [7,33]. The IPDT was composed of Formula (1) [32,33,34,35] as follows:

(1) $$\mathrm{IPDT}={\;A}^{*}K^{*}\;\times \;(T_{f}-T_{i})+T_{i}$$

where A* and K* are the area ratios defined by the TGA analysis curves, T_f_ is the final experimental temperature, and T_i_ is the initial experimental temperature. In this study, T_f_ is 600 °C and T_i_ is 100 °C. Respectively, A* and K* are calculated by Equations (2) and (3), where the values of S_1_, S_2_, and S_3_ given in Figure 8 determine the following:

(2) $$A^{*}=\frac{S_{1}+S_{2}}{S_{1}+S_{2}+S_{3}}$$

(3) $$K^{*}=\frac{S_{1}+S_{2}}{S_{1}}$$

As indicated in Table 5 and Figure 7, the *T*_5%_ of the WZ-cn/GF composites exceeded 405 °C and the *T*_10%_ exceeded 490 °C. The residual carbon ratio (*Yc*) at 600 °C also exceeded 82%, suggesting that the composite laminates possess favorable thermal stability. As the curing temperature rose, the *T*_5%_ of the WZ-cn/GF composites increased by 16.01 °C and 10.7 °C, respectively, and the *T*_10%_ increased by 9.87 °C and 13.65 °C, respectively. The increase in *Yc* was relatively small and remained basically stable, without significant variations in temperature changes. This enhancement in thermal stability is attributed to the increase in the crosslink density along with the rise in curing temperature. Through the integrated procedural decomposition temperature (IPDT) analysis, we conducted a detailed evaluation of the thermal stability of the WZ-cn/GF composites treated at various temperatures. The IPDT calculations reveal that the thermal stability of the WZ-cn/GF composites can be effectively enhanced by raising the thermal curing temperature. This is because an increase in the thermal curing temperature can facilitate the formation of a more stable and rich aromatic heterocyclic structure, which is the key factor in determining thermal stability [15]. However, the IPDT of WZ-cn/GF composites decreased after being cured at 280 °C. The reason for the reduction in the temperature can be elucidated from the following aspects: (1) Curing reaction characteristics: The curing behavior of benzoxazine resin and nitrile resin involves ring-opening polymerization of the benzoxazine ring and ring formation polymerization of the nitrile. These reactions reach a critical point at 280 °C (shown in Figure 6), resulting in a change in the thermal stability of the material [6,7]. (2) Molecular structure changes: Small molecules may be released during the curing process of benzoxazine resin. For instance, volatile substances may be produced during the ring-opening polymerization of benzoxazine, which may affect the thermal stability of the material [26]. (3) Intermolecular force changes: The curing of benzoxazine resins and nitrile-based resins may involve alterations in intermolecular forces, such as the formation and breaking of hydrogen bonds, which may affect the thermal stability of the material [22,36]. (4) Thermal properties of the resin: The thermal properties of the benzoxazine resin itself, such as the glass transition temperature (*T_g_*) and thermal decomposition temperature, may change during curing, thereby affecting the IPDT. In summary, the reduction in the IPDT of benzoxazine nitrile resin composites during curing at 280 °C may be attributed to the combined effects of multiple factors such as curing reaction characteristics, molecular structure changes, intermolecular force changes, and the thermal properties of the resin itself. Specific thermal performance data can be found in Table 5.

#### 3.4.2. Dynamic Mechanical Analysis of WZ-cn/GF Composite Laminates

**Figure 9:**
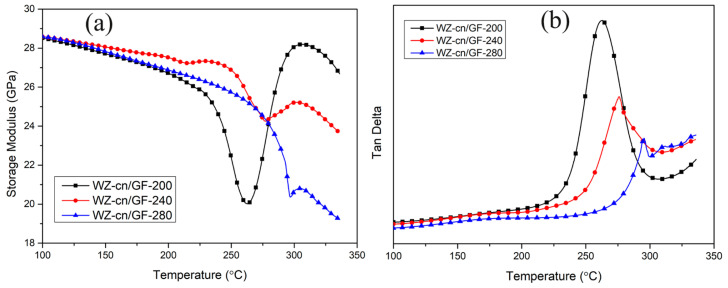
The DMA analysis curves of WZ-cn/GF composite laminates treated at various temperatures: (**a**) storage modulus; (**b**) Tan δ.

The dynamic thermomechanical properties of the composites consisting of WZ-cn/GF were examined. Figure 9a depicts the relationship between the storage modulus of the composites and temperature, while Figure 9b illustrates the variation in the loss tangent of the composites with temperature. In accordance with the theory of glass transition temperature, the peak temperature in Figure 9b is regarded as the *T_g_* (glass transition temperature) of the composites. The crosslinking density of the composites was determined by the DMA method, and the calculation method is presented as Equation (4):

(4) $$\rho =\frac{E^{\prime }}{6\mathrm{RT}}$$

where *ρ* represents the crosslinking density, E′ refers to the storage modulus at the temperature of T (*T_g_* + 40 °C), and R is the vacuum constant. The detailed data are depicted in Table 6.

It can be discerned from Figure 9a that all the composite laminates maintain a high storage modulus at temperatures above 250 °C, which lies within the range of 24 to 28.5 GPa, indicating that both materials exhibit relatively high rigidity. As the curing treatment temperature increases, the initial storage modulus of the system also ascends, which is ascribed to the augmentation of the crosslinking degree of the composites. As the test temperature rises, the storage modulus of the system drops. At higher temperatures, the stress needed to reach this maximum strain gradually decreases, leading to a reduction in sample stiffness and a decrease in the storage modulus [37]. The cause of this phenomenon is that the increase in temperature results in a decline in the binding force between the resin and the fiber, thereby giving rise to a decrease in the storage modulus. When the post-curing treatment temperature is lower than 240 °C, the storage modulus of WZ-cn/GF composites undergoes a second increase, suggesting that in the process of preparing composite materials, due to the obstructive effect of fibers on the molecular flow and crosslinking of the resin, the forming temperature needs to be further elevated in order to obtain a composite with a higher degree of crosslinking [38,39].

As shown in Figure 9b, the increase in treated temperature causes the Tan δ peak to shift to higher temperatures, indicating an increase in *T_g_*. The specific *T_g_* values are presented in Table 6. As the heat treatment temperature rose, the glass transition temperature of the composites significantly increased to 294 °C. Additionally, compared to WZ-cn/GF-200, the Tan Delta peak width for WZ-cn/GF-240 and WZ-cn/GF-280 narrowed, suggesting a more uniform pore size in the composite network and a more refined network structure [40,41].

Based on Table 6 and Formula (4) for calculating crosslinking density, it can be observed that as the curing temperature rises, the crosslinking density of the WZ-cn/GF composites shows a decreasing tendency. This is because the modulus value employed in the formula is chosen when the temperature is 40 °C higher than the glass transition temperature. The composites cured at 240 °C and below undergo secondary curing, that is, the modulus experiences a secondary increase. The modulus value used in the calculation is higher than that of the actual composites, leading to an overestimated calculated crosslinking density result. However, after curing at 280 °C, the secondary modulus increase in the composite did not occur, and the calculated crosslinking density was closer to the actual situation of the composite.

### 3.5. Mechanical Properties of the WZ-cn/GF Composite Laminates

**Figure 10:**
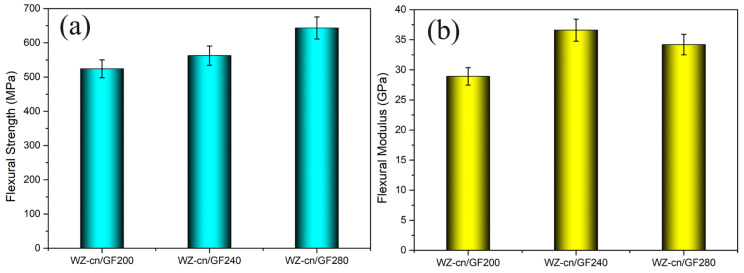
(**a**) Flexural strength and (**b**) flexural modulus of various WZ-cn/GF composite laminates.

Figure 10, respectively, presents the flexural strength and flexural modulus of the composite laminates, and the detailed data are recorded in Table 7. As indicated in Figure 10 and Table 6, all WZ-cn/GF composites exhibit excellent bending strength (520–640 MPa) and bending modulus (28–36 GPa), and the bending modulus of the various composite laminates fluctuates slightly. For the resin matrix composites, the factors that can influence the mechanical properties encompass the resin content in the composite, the molecular structure of the resin matrix, the degree of curing crosslinking of the resin, the type of fiber reinforcement material, and the binding force between the resin and the fiber [37,40,41]. With the increase in the curing temperature, the bending strength of WZ-cn/GF composites rises. This is because of the fact that the degree of the curing reaction of the WZ-cn matrix continues, which makes the matrices display a higher crosslinking degree and density, thereby enhancing the bending strength of the composites.

To explore the impact of the main chain fluorene structure on the structural properties of composite materials, this study prepared fiber-reinforced benzoxazine nitrile-resin with bisphenol A composites (BA-ph/GF) and further disclosed the properties of benzoxazine nitrile-resin containing a fluorene structure by comparing the bending strength and modulus of the two materials. By comparing WZ-cn/GF and BA-ph/GF composites under the same hot-pressing conditions, it can be found that WZ-cn/GF composites possess higher bending strength. The bending strength of WZ-cn/GF-280 is 653 MPa, which is 73 MPa higher than that of BA-ph/GF-280. This might be caused by the following factors. Firstly, compared with the bisphenol structure in BA-ph, the rigid fluorene structure introduced into WZ-cn enhances the rigidity of the molecular chain segment, thus improving the bending resistance of the composite material [16,42]. Secondly, the intermolecular interaction among the rigid chains in WZ-cn molecules is significant, and the steric hindrance is large, resulting in a large activation energy of the flow, making its viscosity change more sensitive to temperature variation [15,16]. As can be seen from the previous section, the initial curing temperature of WZ-cn is higher, and the curing reaction needs to be carried out at a higher temperature. The viscosity of benzoxazinitrile-based resin containing fluorene is at a low level, and it has sufficient wetting time when combined with the fiber, which increases the infiltration of the resin matrix into the fiber. In addition, the introduction of the fluorene-containing structure makes the curing polymerization rate of the resin relatively slow, and it takes a longer time to achieve complete curing at the same temperature. When the curing and polymerization rate of the resin is slow, the gas defects within the composite material are more dissolved and eliminated as the resin remains in a low viscosity state for an extended period. Meanwhile, the resin with lower viscosity can enhance the infiltration effect on the fiber, and further improve the structural properties of the fiber-reinforced composite [18,19].

### 3.6. Micromorphology Analysis of the WZ-cn/GF Composite Laminates

**Figure 11:**
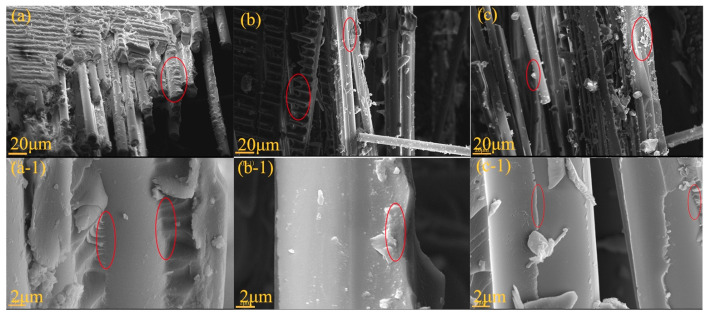
SEM images of the fracture surface of various WZ-cn/GF composite laminates: (**a**) and (**a-1**) WZ-cn/GF-200; (**b**) and (**b-1**) WZ-cn/GF-240; and (**c**) and (**c-1**) WZ-cn/GF-280.

To further investigate the polymerization degree of the WZ-cn matrix and the interface between the fiber and resin matrix of WZ-cn/GF composites, SEM was utilized to monitor the cross-sectional morphology, and the results are presented in Figure 11. It can be observed that when the curing temperature is low, a large area of resin residue can be identified on the fiber surface. Meanwhile, as the curing temperature increases, a stripping phenomenon emerges between the resin matrix and the glass fiber in the cross-section of the composite. When the post-curing temperature reaches 280 °C, the boundary between the resin matrix and the fiber becomes more distinct, and even cracks occur. It can be concluded that with the increase in the curing temperature, the interface strength between the resin matrix and the fiber decreases [43], which is attributed to the fact that at a higher temperature, the high temperature causes the decomposition of uncrosslinked small molecules at the interface of the fiber and the resin matrix, resulting in the presence of cracks.

In comparison with the section topography of the resin loaded on the surface of the fibers shown in Figure 11a-1,b-1,c-1, it can be found that the resin matrix surface cured under high temperature and high pressure has more granular structures and no obvious surface defects. This is caused by the ring polymerization of more nitrile-based resins resulting in the formation of a dense aromatic heterocyclic structure [44]. This indicates that the curing degree of the resin matrix is higher and the crosslinking density is greater, which is also consistent with the analysis results of the thermal stability of the composite material in the subsequent paper. Additionally, there are irregular cracks in the cross-section of the composite resin matrix. This fracture area can significantly increase the fracture path of the resin matrix, enhance the fracture work of the resin matrix, and make the system consume more energy during the fracture process to achieve the purpose of enhancing mechanical properties [37,38].

## 4. Conclusions

In this study, the influence of curing temperature on the polymerization process of the resin matrix and the properties of glass fiber-reinforced resin matrix composites was investigated. The optimal preparation technology of the resin system was determined by DSC and in situ FTIR. DSC time scanning and gelation time testing were employed to compare the effect of different pretreatment conditions on the polymerization process of WZ-cn resin. It was verified that the introduction of fluorene structure augmented the steric hindrance between molecular chains, resulting in an increased difficulty in the polymerization between active functional groups. Subsequently, the fiber-reinforced resin matrix composites were fabricated by solution preimpregnation combined with the hot-pressing method, and the effects of fluorene structure introduction and different post-curing temperatures on the properties of the composites were compared. Finally, the effects of different curing temperatures on the properties of WZ-cn/GF composites were examined through thermal performance testing, mechanical performance testing, and cross-section morphology analysis. The main conclusions are as follows: (1) The increase in the post-curing temperature is beneficial to the curing reaction. The introduction of a fluorene-containing structure makes the movement of WZ-cn molecular segments more arduous and increases the difficulty of the subsequent curing reaction. (2) The increase in post-curing temperature further enhances the curing crosslinking density of the system, thereby improving the glass transition temperature and thermal stability. The *T*_5%_ of WZ-cn/GF rises from 407.4 °C to 423.41 °C, the *T*_10%_ increases from 501.24 °C to 511.11 °C, and the IPDT value increases by 412 °C. (3) The structural strength of the composite material also depends on the variation in curing temperature, and the bending strength and modulus are also improved with the increase in curing temperature. The bending strength of WZ-cn/GF increases from 522 MPa to 653 MPa, and the bending modulus remains within the range of 28 to 37 GPa.

## Figures and Tables

**Table 1:** The heat-press programs of various WZ-cn/GF composite laminates.

Samples	WZ-cn/GF-200	WZ-cn/GF-240	WZ-cn/GF-280
The heat-press program	200 °C-2 h	200 °C-2 h–240 °C-2 h	200 °C-2 h–240 °C-2 h–280 °C-2 h

**Table 2:** DSC data of various WZ-cn pre-polymers.

Samples	*T_i_* (°C)	*T_p_*_1_ (°C)	*T_p_*_2_ (°C)	ΔH_1_ (J/g)	ΔH_2_ (J/g)
WZ-cn	222.5	243.4	277.8	80.4	28.2
WZ-cn-30	237.6	261.3	297.6	72.9	21.3
WZ-cn-60	236.1	262.1	298.0	70.7	17.0
WZ-cn-90	235.8	262.6	299.9	62.4	13.4

**Table 3:** GPC results of various WZ-cn pre-polymers.

Samples	*M_z_*	*M_n_*	*M_w_*	Dispersity Đ	Degree of Polymerization
WZ-cn	868	868	-	-	-
WZ-cn-30	3841	1715	2386	1.61	1.98
WZ-cn-60	5469	1879	3019	1.81	2.16
WZ-cn-90	7636	1953	3689	2.07	2.25

**Table 4:** Gelation time of various WZ-cn pre-polymers.

Samples	WZ-cn	WZ-cn-30	WZ-cn-60	WZ-cn-90
Gelation time (s)	988	754	703	600

**Table 5:** Thermal properties of various Wz-cn/GF composite laminates.

Samples	*T*_5%_ (°C)	*T*_10%_ (°C)	*Y_C_* (%, 600 °C)	IPDT		
				A*	K*	T (°C)
WZ-cn/GF-200	407.4	501.24	83.69	0.9554	8.0611	4286
WZ-cn/GF-240	411.21	500.57	84.71	0.9544	8.8923	4718
WZ-cn/GF-280	423.41	511.11	84.69	0.9547	8.8527	4698

**Table 6:** *T_g_*s and crosslinking degree of the various WZ-cn/GF composites.

Samples	WZ-cn/GF-200	WZ-cn/GF-240	WZ-cn/GF-280
*T_g_* (°C)	262.5	275.36	294.55
Crosslinking degree (mol/m^3^)	0.67 × 10^6^	0.53 × 10^6^	0.43 × 10^6^

**Table 7:** Mechanical properties of WZ-cn/GF and BA-ph/GF composite laminates.

Samples	Flexural Strength (MPa)	Flexural Modulus (GPa)
WZ-cn/GF-200	522	28.9
WZ-cn/GF-240	568	37.5
WZ-cn/GF-280	653	33.8
BA-ph/GF-200	520	25.2
BA-ph/GF-240	557	29.1
BA-ph/GF-280	580	27.8

## Data Availability

The raw/processed data required to reproduce these findings cannot be shared at this time as the data also form part of an ongoing study.
